# 

**Published:** 1858-09

**Authors:** 


### CONTENTS.

ORIGINAL COMMUNICATIONS— PAGE.

Report of Thirteen Cases of Ununited Fracture, treated by Sub-Cutaneous Perforation of the Bone. By Daniel Brainard, M. D....... 421

Resection of Two and a Half Inches of the Os Humeri, the same extent of the Upper End of the Radius, and nearly all the Ulna. By Daniel Brainard, M D.......... 435

Two Cases of Hepatic Abscess, treated in the Mercy Hospital by W. H. Byford, M. D.......... 439

Report of a Foreign Body in the Œsophagus, and its Transit through the Alimentary Canal. By J. W. Redden, M. D.......... 447

Inflammation, Ulceration and Induration of the Os and Cervix Uteri, and Use of the Speculum. By W. M. Chambers, M. D.......... 451

BOOK AND PAMPHLET NOTICES—

Bucknill and Tuke on Insanity.......... 464

A Practical Treatise on the Causes. Symptoms and Treatment of Spermatorrhea. By M. Lallemand.......... 465

PROCEEDINGS OF MEDICAL SOCIETIES—

Meeting of the McLean Co. Medical Society.......... 469

Organization of Medical Society of Mercer Co., Ill.......... 470

EDITORIAL—

"The Senior Editor of the Chicago Medical Journal Feeling Badly!".... 472

Illinois State Medical Society.......... 475

Rush Medical College.......... 476

Shelby Medical College.......... 476

Resignations.......... 477

Obituary.......... 477

Medical Charities.......... 477

Medical Journal Changes.......... 478

New York Quarantine Establishment.......... 478

Number of Medical Colleges in the United States.......... 478

